# Digestibility of amino acids in fish meal and blood-derived protein sources fed to pigs

**DOI:** 10.5713/ab.21.0437

**Published:** 2022-01-05

**Authors:** Chan Sol Park, Olayiwola Adeola

**Affiliations:** 1 Department of Animal Sciences, Purdue University, West Lafayette, IN 47907, USA

**Keywords:** Amino Acid, Blood Product, Digestibility, Fish Meal, Protein, Swine

## Abstract

**Objective:**

An experiment was conducted to determine the standardized ileal digestibility (SID) of amino acids (AA) in fish meal (FM) and blood-derived protein sources including spray-dried porcine plasma (SDPP), porcine red blood cells (PRBC), and blood meal (BM) fed to growing pigs.

**Methods:**

Ten barrows (mean initial body weight of 22.1±1.54 kg) surgically fitted with T-cannulas at the distal ileum were allotted to a duplicated 5×4 incomplete Latin square design with 5 experimental diets and 4 periods. Four experimental diets were prepared to contain FM, SDPP, PRBC, or BM as the sole source of nitrogen. A nitrogen-free diet was prepared and included to estimate the basal ileal endogenous losses of AA. For the 7-day experimental period, pigs were fed for 5 days as adaptation, and ileal digesta samples were collected for 9 hours on days 6 and 7.

**Results:**

The SID of crude protein in BM (48.0%) was less (p<0.05) than in FM, SDPP, and PRBC (83.4%, 83.9%, and 87.3%, respectively). Pigs fed the diet containing BM had less (p<0.05) SID of AA, except isoleucine and proline, than those fed the diet containing FM, SDPP, or PRBC. Among FM, SDPP, and PRBC, there was no difference in the SID of crude protein and all AA, except isoleucine. The SID of isoleucine in PRBC and BM (62.7% and 48.3%, respectively) was less (p<0.05) than in FM and SDPP (88.0% and 84.9%, respectively). The SID of lysine in FM, SDPP, PRBC, and BM was 85.4%, 84.9%, 89.7%, and 51.9%, respectively.

**Conclusion:**

The SID of most AA was not different among FM, SDPP, and PRBC, but BM had lower SID of most AA than FM, SDPP, and PRBC.

## INTRODUCTION

Protein sources derived from animal byproducts have been used in the modern swine production system, especially for weanling pigs because of their high concentrations of digestible crude protein (CP) and amino acids (AA) as well as functional components. In addition, weanling pigs are susceptible to anti-nutritional factors in soybean meal such as glycinin and β-conglycinin, leading to a limited inclusion in diets immediately after weaning [1]. Thus, fish meal (FM) has been widely used in diets for weanling pigs which contains high concentrations of CP and AA as well as macrominerals such as calcium and phosphorus [2]. However, the concentration of AA as well as their digestibility vary among sources of FM depending on the quality of fish materials and processing conditions [3].

During the meat processing, blood from slaughtered animals can be collected, processed, and dried to produce blood meal (BM), which can be used as a protein source in swine diets. On the other hand, collected blood can be treated with anticoagulant and centrifuged to separate plasma, which can be further processed to produce spray-dried animal plasma (SDAP) [4]. It has been reported that dietary supplementation of SDAP improves growth performance by providing immunoglobulins in the intestinal lumen, which bind to the pathogens and reduce the proinflammatory responses and post-weaning diarrhea of pigs [5]. Red blood cells are the byproduct from the production of SDAP, which can be also used in weanling pig diets as a protein source [6]. Red blood cells contain high concentration of CP and lysine but low concentration of isoleucine [7]. Because the major components consisting of blood-derived protein sources are different from each other, digestibility of AA may vary among blood-derived protein sources. However, there is a limited information regarding the comparison of AA digestibility among blood-derived protein sources. Therefore, this study aimed to determine the standardized ileal digestibility (SID) of CP and AA in FM, spray-dried porcine plasma (SDPP), porcine red blood cells (PRBC), and BM fed to growing pigs. The null hypothesis of this study was that the SID of CP and AA are not different among FM and 3 blood-derived protein sources.

## MATERIALS AND METHODS

### Animal care

Protocols of the animal experiment were reviewed and approved by the Purdue University Animal Care and Use Committee (West Lafayette, IN, USA).

### Animals, housing, and experimental design

A total of 10 barrows were surgically fitted with T-cannulas at the distal ileum based on the method reported by Dilger et al [8], followed by 7 days of recovery period. Thereafter, pigs with initial body weight (BW) of 22.1±1.54 kg were moved to metabolism crates (1.22×1.22 m^2^). Pigs were divided into 2 replicates based on BW (i.e., heaviest 5 pigs and lightest 5 pigs) and allotted to a duplicated 5×4 incomplete Latin square design with 5 experimental diets and 4 periods. Pigs had free access to water via nipple drinkers.

### Experimental diets, feeding, and sample collection

Five experimental diets were prepared based on cornstarch and sucrose (Table 1). Four diets were prepared to contain FM, SDPP, PRBC, or BM as the sole source of nitrogen (N) with providing 160 g/kg CP in each diet. Fish meal used in this experiment was mechanically extracted from menhaden and purchased from a local supplier. Blood-derived protein sources in this experiment were obtained from Darling Ingredient Inc. (Cold Spring, KY, USA). Nitrogen-free diet (NFD) was prepared to estimate the basal ileal endogenous losses (BEL) of CP and AA in pigs. Soybean oil and cellulose were each added at 50 g/kg to supply energy and dietary fiber, respectively. Experimental diets were formulated to meet or exceed the vitamin and mineral requirement estimates suggested in National Research Council (NRC) [9]. Chromic oxide was added to all diets at 5 g/kg as an index marker.

Individual BW of pigs were measured at the beginning of each experimental period to calculate the daily amount of feed offered which was 4% of mean BW within replicates. Pigs were fed twice a day at 0800 and 1700 hours. For the 7-day experimental period, pigs were fed for 5 days as adaptation, and ileal digesta samples were collected for 9 hours on days 6 and 7. Ileal digesta samples were collected via T-cannulas by attaching plastic sample bags (Whirl-Pak bag; NASCO, Fort Atkinson, WI, USA) containing 10 mL of 10% formic acid. Attached plastic samples bags were changed every 30 minutes and immediately stored at −20°C. At the end of each experimental period, frozen ileal digesta samples were slightly thawed, pooled within pigs, and subsampled. Collected subsamples were stored at −20°C before further analyses.

### Chemical analyses

Frozen ileal digesta samples were freeze dried before the chemical analyses. Test ingredients, experimental diets, and freeze-dried ileal digesta samples were finely ground (<0.75 mm) using a centrifugal grinder (ZM 200; Retsch GmbH, Haan, Germany). The concentrations of dry matter (DM) in ground ingredients, diets, and freeze-dried ileal digesta samples were analyzed by drying at 105°C for 24 h in a forced-air drying oven (Precision Scientific Co., Chicago, IL, USA; method 934.0) [10]. A combustion method was used to analyze the concentrations of N in ground samples (TruMac N; LECO Corp., St. Joseph, MI, USA; method 990.03) [11], and the concentration of CP was calculated by multiplying the N concentration by 6.25. To digest samples for the analysis of AA, samples of test ingredients, experimental diets, and ileal digesta were hydrolyzed by 6 M HCl (or BaOH for tryptophan analysis) at 110°C for 24 h under N atmosphere. An oxidation of samples using performic acid was conducted for the analysis of methionine and cysteine before digestion. High-performance liquid chromatography was used to determine the concentrations of AA in digested samples after postcolumn derivatization (method 982.30 E [a, b, c]) [10]. The analysis of AA was conducted at University of Missouri Agricultural Experiment Station Chemical Laboratories (Columbia, MO, USA). The concentrations of acid-hydrolyzed ether extract (AEE; method 954.02) [10] and ash (method 942.05) [10] in test ingredients were analyzed. Experimental diets and ileal digesta were analyzed for the concentration of chromium (Cr) using a spectrophotometer (Spark 10M; Tecan Group Ltd., Männedorf, Switzerland) at 450 nm of absorbance after the wet digestion in nitric acid and 70% perchloric acid [12].

### Calculations

The apparent ileal digestibility (AID) of CP and AA in test ingredients were calculated by the index method suggested in Kong and Adeola [13]:


$$\text{AID }(\%)=100\times [1-({\text{Cr}}_{\text{in}}/{\text{Cr}}_{\text{out}})\times ({\text{AA}}_{\text{out}}/{\text{AA}}_{\text{in}})],$$

where Cr_in_ and Cr_out_ represent the concentration of Cr (g/kg DM) in experimental diets and ileal digesta, respectively; AA_in_ and AA_out_ represent the concentration of CP or AA (g/kg DM) in experimental diets and ileal digesta, respectively. Data from pigs fed NFD were used to estimate the BEL [g/kg DM intake (DMI)] of CP and AA using the following equation:


$$\text{BEL }(\text{g}/\text{kg DMI})={\text{AA}}_{\text{out}}\times ({\text{Cr}}_{\text{in}}/{\text{Cr}}_{\text{out}}).$$

To calculate the SID of CP and AA in test ingredients, the AID of CP and AA were corrected for the estimated BEL of CP and AA using the following equation:


$$\text{SID }(\%)=\text{AID}+[100\times (\text{BEL}/{\text{AA}}_{\text{in}})].$$

### Statistical analyses

Data were tested for normality by univariate procedure of SAS (version 9.4; SAS Inst. Inc., Cary, NC, USA), and data outside of 2.5 times interquartile range were considered outliers. Thereafter, data were analyzed by mixed linear models procedure of SAS. Model included experimental diet as fixed variable and replicate, period within replicate, and pig within replicate as random variables. Pairwise comparison with the Tukey’s adjustment was conducted to separate the estimated least squares means among experimental diets. Experimental unit was the individual pig. Significance of the model and difference was determined by p<0.05.

## RESULTS

All pigs were healthy throughout except one pig fed NFD during the last experimental period, which was removed from experiment. Also, data from 2 pigs fed NFD and 1 pig fed the diet containing PRBC were detected as outliers and therefore treated as missing observations in the dataset.

On an as-fed basis, the concentration of CP in test ingredients ranged from 658 g/kg for FM to 968 g/kg for PRBC (Table 2). The concentration of AEE in SDPP was 26.2 g/kg, whereas that in PRBC was 8.3 g/kg. Fish meal had the greatest concentration of ash at 201.4 g/kg. The analyzed concentrations of AA in experimental diets are close to the calculated values based on the analyzed concentrations of AA in test ingredients (Table 3).

The AID of CP and AA, except isoleucine, in BM were less (p<0.05) than in the other test ingredients (Table 4). There was no difference in the AID of AA among FM, SDPP, and PRBC, except isoleucine, methionine, and cysteine. Pigs fed the diets containing PRBC and BM had less (p<0.05) AID of isoleucine than those fed the diets containing FM and SDPP. The AID of methionine in FM was greater (p<0.05) than in SDPP, but not different from the value in PRBC. Pigs fed the SDPP diet had greater (p<0.05) AID of cysteine than those fed the PRBC diet, which was not different from FM.

The BEL of CP in pigs fed NFD was 19.9 g/kg DMI (Table 5). The BEL of indispensable AA ranged from 89 mg/kg DMI for methionine to 666 mg/kg DMI for valine. The SID of CP in BM was less (p<0.05) than in FM, SDPP, and PRBC (Table 6). Pigs fed the diet containing BM had less (p<0.05) SID of AA, except isoleucine and proline, than those fed the diet containing FM, SDPP, or PRBC. Among FM, SDPP, and PRBC, there was no difference in the SID of CP and all AA, except isoleucine. The SID of isoleucine in PRBC and BM was less (p<0.05) than in FM and SDPP.

## DISCUSSION

The analyzed CP, AA, and ash concentrations in FM are consistent with the previously reported values [9,14,15]. In addition, the concentration of AEE in FM also agrees with the values reported in Casas et al [14] and Lagos and Stein [15]. The FM used in the current study is representative due to similarity in AA concentration for FM reported in previous studies [9,14,15]. The concentration of AA in SDPP are comparable to the values for plasma protein [9] and the values for SDAP [16] despite the differences in source of blood plasma. Torrallardona [5] reported that the concentration of AA in SDPP was not substantially different from that in spray-dried bovine plasma (SDBP). However, SDPP used in the current study has greater indispensable AA and lower ash concentrations compared to SDPP used in Wu et al [17], which may be due to the differences in processing conditions [5]. Compared to nutrient composition of SDAP in previous studies [9,16,17], SDPP used in the current study contains slightly greater CP and lower ash. This observation is consistent with Torrallardona [5] who suggested that the concentration of CP in SDAP is negatively related to the concentration of ash. The concentration of indispensable AA in PRBC agrees with the values for spray-dried blood cells [6,16]. Because blood cells are almost exclusively composed of hemoglobin [4], it is assumed that nutrient compositions of blood cells products are relatively consistent among sources regardless of species origins. The concentration of CP in BM is slightly greater than previously reported CP concentrations ranging from 887 to 917 g/kg as-fed basis, whereas the concentration of indispensable AA, except isoleucine and leucine, is close to the previously reported values [9,16,18]. Compared to the reference values, the concentration of isoleucine was greater, but that of leucine was lower in BM used in the current study. This may be due to the reduced inclusion rate of blood cells because blood cells contain low concentration of isoleucine and high concentration of leucine. In addition, BM used in the current study was produced using a mixture of blood collected from both beef and pork productions, and therefore, variations in AA concentrations might be related to the ratio between bovine and porcine blood.

Fish meal and SDPP have been widely used in diets for weanling pigs due to their high quality of protein and favorable AA contents for animal growth [2,19]. Although the use of PRBC in swine diets is not as common as FM or SDPP, practical applications in weanling pig diets have been reported in previous publications [6,7,20]. Even though animal protein sources have been generally used for weanling pigs, they have been also used in growing pig diets depending on the availability of feed ingredients or functional purposes. Therefore, the current experiment was conducted to determine the SID values using growing pigs to provide necessary information required to properly use FM and blood-derived protein sources in diets for growing pigs.

The SID of CP and AA, except isoleucine, were not different among FM, SDPP, and PRBC, which may indicate that FM, SDPP, and PRBC tested in this experiment have similar AA availability for pigs. However, it should be noted that the functional properties of FM, SDPP, and PRBC are different when fed to weanling pigs despite the similarity of SID of AA. Fish meal has been used in weanling pig diets mainly because it contains highly digestible and balanced AA as well as poly unsaturated fatty acids [2] and because it is a cost-effective protein source compared to SDPP. On the other hand, SDPP has been added in diets for weanling pigs to improve the immune status of pigs by providing exogenous immunoglobulins, leading to the reduction of post-weaning diarrhea and the increase in growth performance [5]. Therefore, together with the SID of AA in FM and SDPP, their functional properties should be considered when formulating diets containing FM and SDPP. The reason for less SID of isoleucine in PRBC compared to FM and SDPP remains unclear; perhaps this is partly due to the low concentration of isoleucine (5.8 g/kg as-fed basis) in PRBC, which translates to less than 1 g/kg of diet. Such a low dietary concentration is susceptible to attendant analytical errors. This observation implies that crystalline isoleucine or isoleucine-rich feed ingredient is required to prevent potential isoleucine deficiency when feeding PRBC to pigs.

The BEL of CP and AA observed in this study are comparable to the values summarized in previous studies [9,21,22]. The BEL of proline was relatively greater than the BEL of other AA, which may be due to the altered AA metabolism in the gastrointestinal tract caused by deficiency of AA in pigs fed NFD [22]. The SID of CP and AA in FM are close to the SID values of menhaden FM reported in Rojas et al [23] and Casas et al [14], but somewhat greater than SID values of menhaden FM presented by Lagos and Stein [15]. This inconsistency may be due to the differences in BW of pigs used in experiments or FM products such as processing conditions or oxidation [3]. In addition, Jones et al [24] reported that the inclusion rate of fish solubles in FM may influence the nutritional values of FM, although growth performance was not affected by the inclusion of fish solubles.

The SID of CP and AA in SDPP observed in the current study are in agreement with NRC [9] and Wu et al [17]; however, the SID values in SDPP are less than the values reported in Gottlob et al [25] and Almeida et al [16], in which SDAP was used as a test ingredient. In a meta-analysis study reported by Balan et al [19], there was no difference between SDPP and SDBP in growth performance of weanling pigs, but weanling pigs fed SDAP had lower growth performance than those fed SDPP and SDBP. However, it should be noted that beneficial effect of feeding plasma protein is likely due to its immunoglobulin content, which may mitigate the inflammation of weanling pigs [5]. In other words, greater beneficial effect of SDPP compared to SDAP may not indicate the greater digestibility of AA in SDPP than SDAP. Further research is needed to systemically compare the overall nutritional quality of plasma protein sources focusing on digestible AA contents and bioactive proteins (i.e., immunoglobulins), all of which directly influence the growth performance of weanling pigs.

The information for the SID of CP and AA in PRBC as well as blood cells is scarce, but the SID of CP and AA in PRBC are less than those in spray-dried blood cells reported by Almeida et al [16]. This difference is likely due to variations in processing of blood cells after centrifugation. Because the concentration of AA in PRBC is close to those reported by Almeida et al [16], it is assumed that the high temperature during the drying process might negatively affect proteins and AA in PRBC, resulting in a slight reduction of SID of AA. However, the potential heat damage on PRBC may not be as severe as on BM in the current study.

Several processes in the production of BM, such as heat treatment, pH control, or filtration, may negatively affect the availability of protein and AA [4]. In addition, high temperature during the drying process may cause the Maillard reaction which reduces the digestibility of lysine in feed ingredients [26]. Therefore, it is speculated that proteins in BM used in the current study was damaged during the production process, resulting in lower SID of most AA than FM as well as the other blood-derived sources. Due to the high BEL of proline and reduced digestibility by heat damage, the SID of proline was considerably lower than the other AA. Moreover, the SID of CP and AA in BM observed in the current study were lower than the values reported in the previous studies [9,16,18], which may be another evidence of protein denaturation in BM.

## CONCLUSION

Based on the results of this experiment, the null hypothesis of this study was rejected, and it was concluded that the SID of CP and most AA in BM were less than in FM, SDPP, and PRBC. The SID of CP and AA, except isoleucine, were not different among FM, SDPP, and PRBC. The SID of isoleucine in PRBC and BM was less than in FM and SDPP. This study revealed that FM, SDPP, and PRBC have similar digestibility of AA, except isoleucine, all of which contain high quality AA balance to support the optimal growth of pigs.

## Figures and Tables

**Table 1:** Ingredient composition of experimental diets containing fish meal, spray-dried porcine plasma, porcine red blood cells, and blood meal (g/kg as-fed basis)

Ingredient	FM	SDPP	PRBC	BM	NFD
Corn starch	320.0	345.2	367.3	363.3	316.0
Sucrose	300.0	300.0	300.0	300.0	500.0
Fish meal	246.0	0.0	0.0	0.0	0.0
Spray-dried porcine plasma	0.0	191.0	0.0	0.0	0.0
Porcine red blood cells	0.0	0.0	164.0	0.0	0.0
Blood meal	0.0	0.0	0.0	168.0	0.0
Soybean oil	50.0	50.0	50.0	50.0	50.0
Cellulose^1)^	50.0	50.0	50.0	50.0	50.0
Ground limestone	0.0	17.8	13.0	13.0	13.0
Monocalcium phosphate	0.0	12.0	21.7	21.7	23.5
Salt	4.0	4.0	4.0	4.0	0.0
Potassium carbonate	0.0	0.0	0.0	0.0	2.6
Magnesium oxide	0.0	0.0	0.0	0.0	2.0
Sodium bicarbonate	0.0	0.0	0.0	0.0	7.5
Choline chloride	0.0	0.0	0.0	0.0	2.5
Potassium chloride	0.0	0.0	0.0	0.0	2.9
Vitamin-mineral premix^2)^	5.0	5.0	5.0	5.0	5.0
Chromic oxide premix^3)^	25.0	25.0	25.0	25.0	25.0
Total	1,000.0	1,000.0	1,000.0	1,000.0	1,000.0

FM, fish meal; SDPP, spray-dried porcine plasma; PRBC, porcine red blood cells; BM, blood meal; NFD, nitrogen-free diet.

1) Solka-Floc 40 FCC (International Fiber Corporation, Urbana, OH, USA).

2) Provided the following quantities per kilogram of complete diet: vitamin A, 8,575 IU; vitamin D_3_, 4,300 IU; vitamin E, 28.6 IU; menadione, 7.30 mg; riboflavin, 9.15 mg; D-pantothenic acid, 18.3 mg; niacin, 73.5 mg; choline chloride, 1,285 mg; vitamin B_12_, 0.02 mg; biotin, 0.09 mg; thiamine mononitrate, 3.67 mg; folic acid, 1.65 mg; pyridoxine hydrochloride, 5.50 mg; I, 1.85 mg as ethylenediamine dihydroiodide; Mn, 180 mg as manganous oxide; Cu, 7.40 mg as copper sulfate; Fe, 73.5 mg as ferrous sulfate; Zn, 180 mg as zinc oxide; and Se, 0.43 mg as sodium selenite.

3) Prepared by adding 5 g chromic oxide to 20 g sucrose.

**Table 2:** Analyzed concentration of nutrients and gross energy in fish meal, spray-dried porcine plasma, porcine red blood cells, and blood meal (g/kg as-fed basis)

Nutrient	FM	SDPP	PRBC	BM
Dry matter	911	936	896	911
Gross energy (MJ/kg)	18.3	20.2	22.0	22.4
Crude protein^1)^	658	826	968	944
Acid-hydrolyzed ether extract	86.9	26.2	8.3	20.3
Ash	201.4	72.7	9.8	22.5
Indispensable amino acid				
Arginine	36.6	47.4	39.0	48.6
Histidine	12.2	26.7	73.7	49.6
Isoleucine	25.5	30.3	5.8	36.7
Leucine	41.8	78.3	129.0	96.5
Lysine	47.1	69.0	81.9	76.6
Methionine	16.9	6.9	7.2	11.7
Phenylalanine	24.3	46.6	65.5	55.9
Threonine	24.5	45.8	28.8	43.0
Tryptophan	6.2	14.9	14.8	9.0
Valine	29.2	55.0	89.4	61.0
Dispensable amino acid				
Alanine	39.7	43.5	77.6	67.0
Aspartic acid	53.6	76.2	110.0	81.5
Cysteine	5.1	27.6	6.9	14.1
Glutamic acid	77.8	113.4	75.2	87.7
Glycine	48.6	28.6	44.7	34.7
Proline	29.5	48.8	33.4	37.4
Serine	21.4	42.5	37.1	34.6
Tyrosine	17.2	42.3	20.3	30.8

FM, fish meal; SDPP, spray-dried porcine plasma; PRBC, porcine red blood cells; BM, blood meal.

1) Nitrogen×6.25.

**Table 3:** Analyzed concentration of dry matter, crude protein, and amino acids in experimental diets containing fish meal, spray-dried porcine plasma, porcine red blood cells, and blood meal (g/kg as-fed basis)

Nutrient	FM	SDPP	PRBC	BM	NFD
Dry matter	952	948	945	948	963
Crude protein^1)^	180	170	166	154	11
Indispensable amino acid					
Asrginine	9.2	8.8	5.9	7.4	0.0
Histidine	3.1	5.0	10.5	7.9	0.0
Isoleucine	6.7	6.0	1.3	6.2	0.0
Leucine	10.9	15.3	19.4	16.1	0.1
Lysine	12.3	13.5	12.8	12.5	0.1
Methionine	4.3	1.3	1.1	1.9	0.1
Phenylalanine	6.2	9.1	10.0	9.2	0.1
Threonine	6.2	8.8	4.8	7.0	0.0
Tryptophan	1.7	2.9	1.9	0.8	0.0
Valine	7.8	10.9	13.5	10.1	0.1
Dispensable amino acid					
Alanine	10.6	8.7	11.6	11.0	0.1
Aspartic acid	14.0	15.0	16.9	13.5	0.1
Cysteine	1.3	5.4	1.4	2.3	0.0
Glutamic acid	20.8	22.6	12.7	14.5	0.1
Glycine	12.8	5.7	6.9	5.7	0.1
Proline	8.0	9.2	5.3	5.7	0.2
Serine	5.3	7.8	5.9	5.7	0.1
Tyrosine	3.8	6.2	2.7	3.9	0.0

FM, fish meal; SDPP, spray-dried porcine plasma; PRBC, porcine red blood cells; BM, blood meal; NFD, nitrogen-free diet.

1) Nitrogen×6.25.

**Table 4:** Apparent ileal digestibility (%) of crude protein and amino acids in fish meal, spray-dried porcine plasma, porcine red blood cells, and blood meal fed to growing pigs^1)^

Item	FM	SDPP	PRBC	BM	SEM	p-value
Crude protein	72.8^a^	72.8^a^	76.0^a^	35.8^b^	3.43	<0.001
Indispensable amino acid						
Arginine	84.1^a^	80.9^a^	78.7^a^	43.5^b^	3.71	<0.001
Histidine	79.5^a^	83.2^a^	88.8^a^	44.3^b^	3.38	<0.001
Isoleucine	83.5^a^	79.9^a^	39.7^b^	43.5^b^	4.43	<0.001
Leucine	83.8^a^	82.8^a^	87.0^a^	43.1^b^	3.18	<0.001
Lysine	81.5^a^	81.3^a^	85.9^a^	48.0^b^	3.14	<0.001
Methionine	86.9^a^	75.1^b^	80.9^ab^	55.0^c^	3.03	<0.001
Phenylalanine	81.2^a^	82.6^a^	86.7^a^	42.2^b^	3.33	<0.001
Threonine	75.9^a^	72.4^a^	69.0^a^	40.4^b^	3.76	<0.001
Tryptophan	84.3^a^	81.8^a^	85.3^a^	44.3^b^	3.74	<0.001
Valine	79.7^a^	75.3^a^	84.6^a^	37.8^b^	3.37	<0.001
Dispensable amino acid						
Alanine	81.1^a^	78.1^a^	86.0^a^	41.6^b^	3.34	<0.001
Aspartic acid	70.9^a^	74.3^a^	83.8^a^	35.6^b^	3.45	<0.001
Cysteine	57.2^ab^	74.4^a^	51.3^b^	24.4^c^	4.91	<0.001
Glutamic acid	81.3^a^	76.6^a^	77.1^a^	42.3^b^	3.51	<0.001
Glycine	70.0^a^	57.1^a^	68.6^a^	20.5^b^	5.46	<0.001
Proline	25.9^a^	42.3^a^	28.4^a^	−73.9^b^	22.56	0.003
Serine	73.5^a^	71.8^a^	77.3^a^	40.7^b^	3.42	<0.001
Tyrosine	80.4^a^	81.4^a^	75.3^a^	44.5^b^	3.93	<0.001

FM, fish meal; SDPP, spray-dried porcine plasma; PRBC, porcine red blood cells; BM, blood meal; SEM, standard error of the mean.

1) Each least squares mean represents 8 observations except for pigs fed PRBC (7 observations).

a–c Within a row, means with different superscripts differ (p<0.05).

**Table 5:** Basal ileal endogenous losses (mg/kg dry matter intake) of crude protein and amino acids in growing pigs fed nitrogen-free diet^1)^

Item	BEL	SD
Crude protein^2)^ (g/kg DMI)	19.9	4.30
Indispensable amino acid		
Arginine	615	187.5
Histidine	187	35.9
Isoleucine	317	41.6
Leucine	566	83.1
Lysine	511	80.0
Methionine	89	16.1
Phenylalanine	338	44.2
Threonine	661	58.7
Tryptophan	111	22.4
Valine	666	47.6
Dispensable amino acid		
Alanine	561	80.7
Aspartic acid	816	103.3
Cysteine	199	22.4
Glutamic acid	989	136.2
Glycine	1459	572.7
Proline	5015	2769.0
Serine	589	61.5
Tyrosine	245	33.5

BEL, basal ileal endogenous loss; SD, standard deviation; DMI, dry matter intake.

1) Each mean represents 5 observations.

2) Nitrogen×6.25.

**Table 6:** Standardized ileal digestibility (%) of crude protein and amino acids in fish meal, spray-dried porcine plasma, porcine red blood cells, and blood meal fed to growing pigs^1)^

Item	FM	SDPP	PRBC	BM	SEM	p-value
Crude protein	83.4^a^	83.9^a^	87.3^a^	48.0^b^	3.43	<0.001
Indispensable amino acid						
Arginine	90.4^a^	87.6^a^	88.5^a^	51.4^b^	3.71	<0.001
Histidine	85.3^a^	86.7^a^	90.5^a^	46.5^b^	3.38	<0.001
Isoleucine	88.0^a^	84.9^a^	62.7^b^	48.3^b^	4.43	<0.001
Leucine	88.8^a^	86.3^a^	89.8^a^	46.5^b^	3.18	<0.001
Lysine	85.4^a^	84.9^a^	89.7^a^	51.9^b^	3.14	<0.001
Methionine	88.9^a^	81.7^a^	88.6^a^	59.5^b^	3.03	<0.001
Phenylalanine	86.4^a^	86.1^a^	89.9^a^	45.7^b^	3.33	<0.001
Threonine	86.1^a^	79.6^a^	82.0^a^	49.3^b^	3.76	<0.001
Tryptophan	90.5^a^	85.4^a^	90.8^a^	56.8^b^	3.74	<0.001
Valine	87.8^a^	81.1^a^	89.3^a^	44.0^b^	3.37	<0.001
Dispensable amino acid						
Alanine	86.1^a^	84.2^a^	90.5^a^	46.4^b^	3.34	<0.001
Aspartic acid	76.5^a^	79.5^a^	88.4^a^	41.3^b^	3.45	<0.001
Cysteine	71.7^a^	77.8^a^	64.7^a^	32.6^b^	4.91	<0.001
Glutamic acid	85.8^a^	80.7^a^	84.5^a^	48.8^b^	3.51	<0.001
Glycine	80.9^a^	81.4^a^	88.6^a^	44.7^b^	5.46	<0.001
Proline	85.6^ab^	94.0^a^	117.9^a^	9.5^b^	22.56	0.009
Serine	84.1^a^	79.0^a^	86.7^a^	50.5^b^	3.42	<0.001
Tyrosine	86.6^a^	85.2^a^	83.9^a^	50.5^b^	3.93	<0.001

FM, fish meal; SDPP, spray-dried porcine plasma; PRBC, porcine red blood cells; BM, blood meal; SEM, standard error of the mean.

1) Each least squares mean represents 8 observations except for pigs fed PRBC (7 observations).

a,b Within a row, means with different superscripts differ (p<0.05).
